# Case Report: Unveiling occult bleeding: a rare case of hemobilia diagnosed by peroral cholangioscopy

**DOI:** 10.3389/fmed.2026.1902918

**Published:** 2026-08-12

**Authors:** Xue Peng, Dengqun Sun, Minghui Sheng, Huilai Luo

**Affiliations:** Department of General Surgery, The Chinese People's Armed Police Forces Anhui Provincial Corps Hospital, Hefei, China

**Keywords:** gallbladder pathology, gastrointestinal hemorrhage, hemobilia, occult bleeding, peroral cholangioscopy

## Abstract

Hemobilia is a relatively rare clinical condition that can potentially cause lifethreatening gastrointestinal hemorrhage. Its non-specific and variable clinical presentation can result in delayed diagnosis or misdiagnosis. This complexity has rendered both the diagnosis and management of such cases a significant challenge in clinical practice. Herein, we present a case of hemobilia where a novel ultra-thin cholangioscope was employed to directly visualize the biliary tree, including the region proximal to the gallbladder, for diagnostic and therapeutic purposes.

## Introduction

Hemobilia, a condition characterized by bleeding into the biliary tract, presents a diverse clinical spectrum. While it can range from self-limited bleeding to potentially life-threatening hemorrhage, its insidious nature and variable symptoms can often lead to delayed diagnosis and underdiagnosis, posing a significant challenge in clinical practice (1). This complexity necessitates a thorough diagnostic approach and tailored management strategies. In this report, we describe a case of hemobilia where a novel ultra-thin cholangioscope (eyeMax; Micro-Tech, Hefei, China) was successfully used for direct visualization of the bilary tree including the cystic duct and the source of hemobilia was identified, which ultimately guided surgical intervention.

## Case report

**Figure 1:**
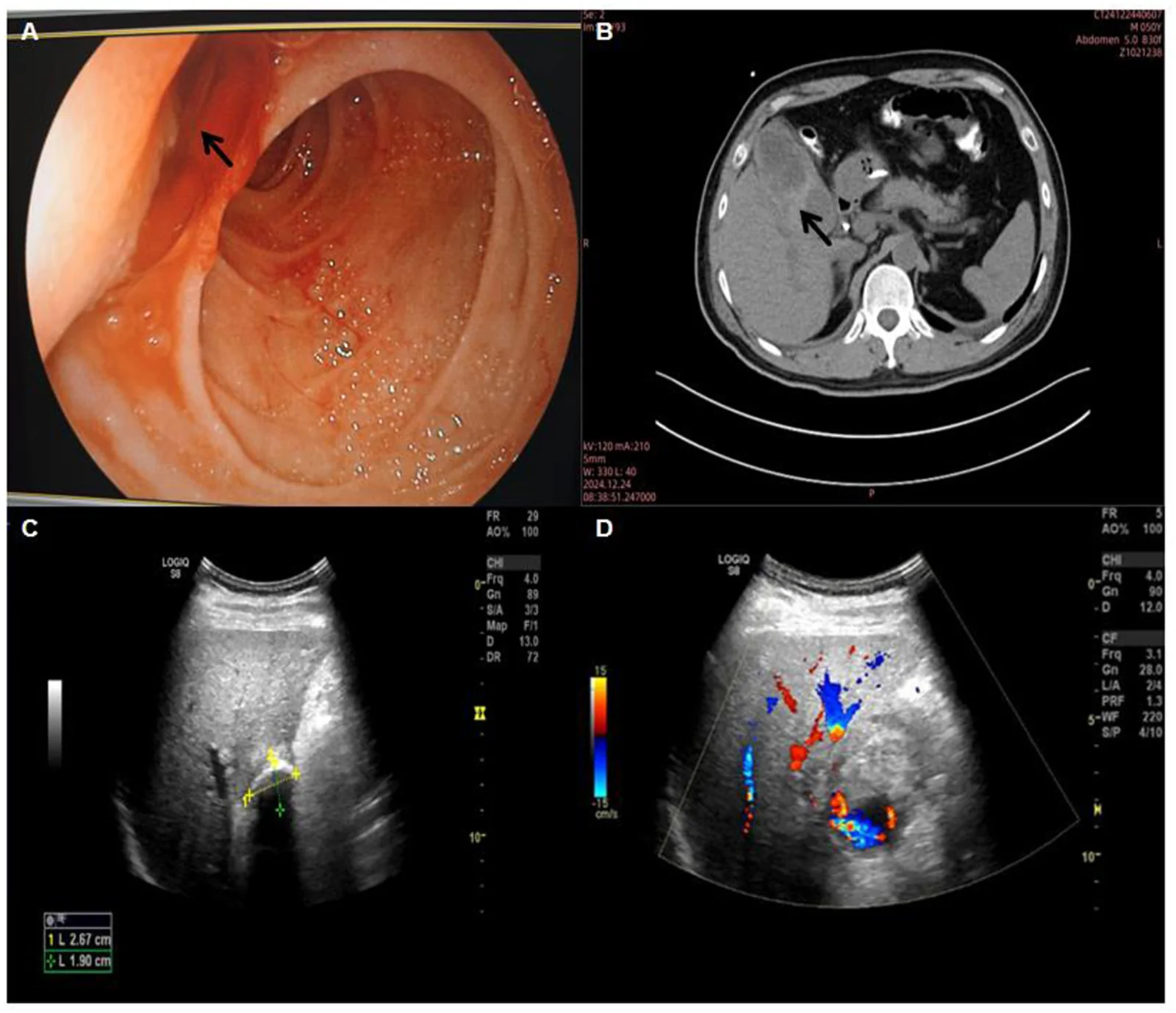
**(A)** Endoscopic examination revealed active oozing from the duodenal papilla (arrow), without a distinct bleeding point; however, bright red refluxing blood was evident. **(B)** Non-contrast computed tomography (CT) demonstrated an enlarged gallbladder with thickened walls and a slightly hyperdense area in the gallbladder (arrow), which may indicate the presence of blood components. **(C)** Ultrasound displayed an irregular anechoic region at the neck of the gallbladder. **(D)** CDFI demonstrated abundant arterial blood flow.

A 50-year-old male presented with intermittent right upper quadrant abdominal pain radiating to the flank and associated with nausea and vomiting of coffee-ground material, raising suspicion of upper gastrointestinal bleeding. On the morning of the day after admission, emergency gastroscopy was performed at 9 a.m. and revealed active ooze from the ampulla of Vater in the descending duodenum (Figure 1A). Subsequently, at 10 a.m., a contrast-enhanced abdominal computed tomography (CT) scan was performed, which demonstrated a patchy area of slightly increased density within the gallbladder (Figure 1B). An ultrasound examination was also conducted on the same morning, indicating an approximately 27 × 20 mm anechoic lesion at the neck of the gallbladder, with an irregular

morphology (Figure 1C). Color Doppler flow imaging (CDFI) revealed rich color flow signals within the lesion, and Doppler studies exhibited an arterial waveform (Figure 1D).

**Figure 2:**
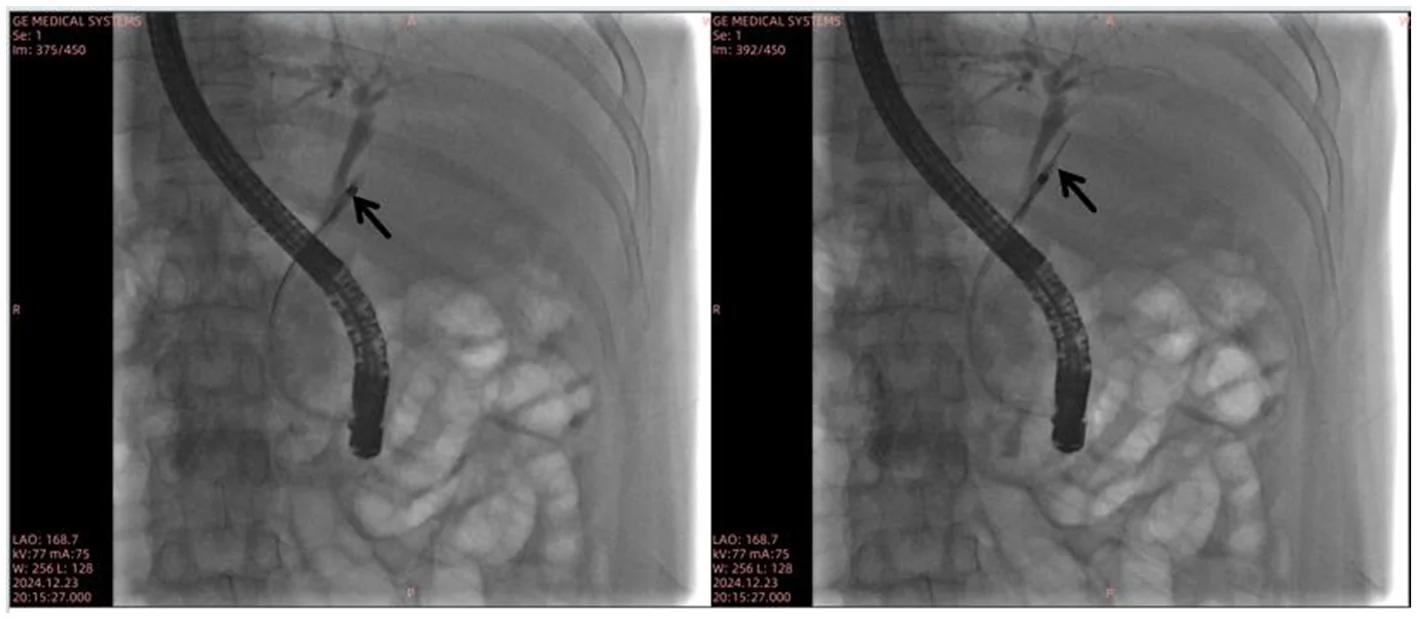
Endoscopic retrograde cholangiopancreatography (ERCP). Cholangiography illustrates the orifice and course of the cystic duct (arrow). The Digital Subtraction Angiography image displays the guidewire and the cannula inserted into the gallbladder.

**Figure 3:**
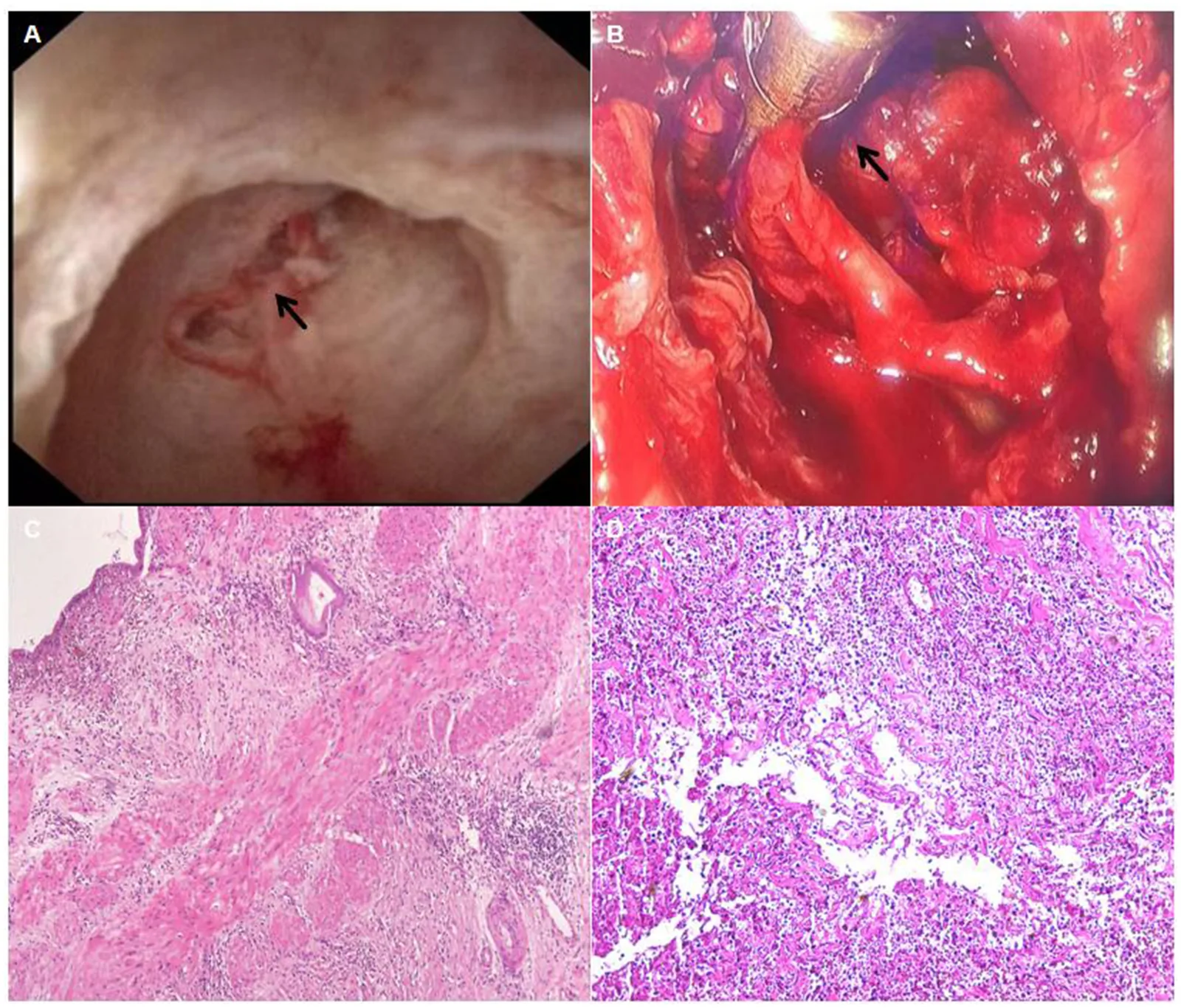
**(A)** Cholangiopancreatoscopy indicated the presence of a floating blood clot within the cystic duct (arrow), and attempts to access the gallbladder via the cystic duct were unsuccessful. **(B)** Laparoscopic examination revealed active vascular bleeding within the perforated gallbladder, characterized by arterial bleeding into the gallbladder lumen. Hemostatic clipping was successfully applied (arrow). (**C, D)** Histological examination of the gallbladder specimen stained with hematoxylin and eosin (H&E) demonstrated areas of thickened gallbladder wall C ( × 10), regions of mucosal hemorrhage, necrosis, and sloughing of fragmented wall tissues. These findings support the diagnosis of intra-biliary hemorrhage D ( × 40).

At 20:30 p.m., Endoscopic retrograde cholangiopancreatography (ERCP) was performed, during which successful cannulation of the biliary tree was achieved. Cholangiography visualized the orifice and trajectory of the cystic duct, and a cholangioscope was advanced (Figure 2). While no bleeding points were identified in the common, hepatic, or peripheral bile ducts, a floating filamentous blood clot was encountered obstructing the cystic duct approximately 1 cm from its origin (Figure 3A). Attempts to advance a guidewire into the gallbladder were unsuccessful. Later, at 1 p.m. on the second day after admission, laparoscopic cholecystectomy was performed.

Preoperative imaging, including ultrasound and computed tomography (CT), provided complementary findings. Ultrasound revealed a distended gallbladder with a thickened, uneven wall, displaying echogenic foci with acoustic shadowing suggestive of gallstones. It also noted an irregular anechoic area at the gallbladder neck, raising suspicion for a pseudoaneurysm. CT corroborated the presence of gallstones with intraluminal patchy high-density areas and demonstrated a distended gallbladder with thickened, edematous walls. The indistinct liver-gallbladder border raised concern for gallbladder perforation. Additionally, intraluminal hyperdensity in the gallbladder lumen was the greatest concern for blood products. Importantly, neither ultrasound, CT, nor ERCP definitively identified gallbladder perforation prior to surgery.

The combined findings of ERCP (cystic duct obstruction by blood clot, inability to visualize gallbladder interior) and imaging (gallstones, gallbladder inflammation with effusion, suspicion of arterial malformation/erosion on ultrasound with possible active bleeding; gallstones, gallbladder wall thickening, and suspicion of perforation on CT) strongly suggested impacted stones at the gallbladder neck with associated hemorrhage. Given the inability to achieve hemostasis endoscopically and the concerning imaging findings, combined with the patient's critical condition but good compliance, laparoscopic cholecystectomy was deemed necessary for both diagnosis and treatment.

Intraoperatively, a perforation of the gallbladder neck with active, significant arterial hemorrhage was confirmed. The bleeding was traced to the cystic artery, and hemostasis was successfully achieved with clamp application (Figure 3B). Postoperative pathology confirmed hemorrhage, necrosis, and sloughing of the gallbladder mucosa, establishing a definitive diagnosis of gallbladder hemorrhage. Histopathologic examination of the gallbladder wall revealed features consistent with severe cholecystitis, accounting for the wall thickening and edema observed on imaging (Figures 3C, D). The postoperative course was uneventful. During follow-up, the patient showed a favorable recovery. At the 3-month follow-up visit, he reported regular bowel movements without recurrent bleeding, melena, or hematemesis. Liver function tests, including transaminases and bilirubin, were within normal limits. Complete blood count also remained normal, with a normal hemoglobin level and no evidence of anemia. Follow-up ultrasonography showed postoperative changes after cholecystectomy, with no dilatation of the intrahepatic or extrahepatic bile ducts and no biliary calculi. At the 6-month follow-up, the patient remained in good condition, and routine physical examination was unremarkable.

In this case, oral cholangioscopy was utilized to visualize the biliary system, providing direct observation of the internal conditions and delineating subtle pathological changes. While the cholangioscope could not be advanced into the gallbladder due to an obstructing blood clot within the cystic duct, and the presence of stones as the direct cause of bleeding remains uncertain, the direct visualization offered valuable diagnostic information regarding the biliary tree. This technique has emerged as a potentially important tool for evaluating conditions within the biliary system. Moreover, this case highlights the importance of a comprehensive diagnostic evaluation and multidisciplinary collaboration, which enhances success rates and improves patient prognosis.

## Discussion

Hemorrhage resulting in upper gastrointestinal bleeding (UGIB) originating from the cystic artery, a phenomenon known as hemobilia, presents a complex and infrequently reported clinical scenario. Under normal physiological conditions, the cystic artery exclusively supplies the gallbladder. However, pathological processes can compromise its integrity, leading to arterial rupture and subsequent bleeding into the biliary tree (1). In cases of gallbladder necrosis, as observed in this patient, a ruptured cystic artery can lead to blood extravasation through the cystic duct and common bile duct into the duodenum, ultimately manifesting as UGIB.

Hemobilia is a rare condition with a multifactorial etiology. Common causes include gallstones, which can induce inflammation, ulceration, and erosion into adjacent blood vessels, particularly the cystic artery (2). Vascular abnormalities, such as aneurysms or pseudoaneurysms of the hepatic or cystic arteries, are another significant contributing factor (2–4). Other less common causes encompass trauma (blunt or iatrogenic), inflammatory conditions like acute cholecystitis, and tumor invasion of blood vessels within or near the biliary tree (5). Arterio-biliary fistula is also a recognized, albeit rare, cause (6). In this particular case, surgical pathology confirmed gallbladder necrosis attributed to bleeding. The presumed underlying mechanism was prolonged compression of gallbladder tissue by impacted stones at the neck, leading to mucosal ischemia, necrosis, and subsequent arterial erosion with possible pseudoaneurysm formation and re-bleeding. This case underscores the critical need for a thorough investigation into potential underlying causes in the management of such rare presentations. In this case, the vascular abnormality may have been related to arterial erosion with pseudoaneurysm formation, although definitive confirmation was not available.

To establish a robust diagnostic reasoning, other potential etiologies were systematically excluded. Hepatic artery or cystic artery pseudoaneurysm was also considered. However, no typical vascular dilatation was identified on CT angiography (CTA), and the bleeding source was ultimately localized to the gallbladder wall. Therefore, a definite diagnosis of pseudoaneurysm could not be established, although arterial erosion with possible pseudoaneurysm formation remained a plausible explanation. Biliary tract malignancy was also evaluated; however, the absence of focal mass lesions on imaging and the characteristic presentation of impacted stones and gallbladder necrosis argued against a neoplastic etiology (7). Traumatic or iatrogenic causes were ruled out based on the patient's history, which lacked any recent procedures or trauma (8). Acute biliary pancreatitis, another potential cause of hemobilia (9), was not evident in this patient's presentation.

The diagnosis of hemobilia can be challenging due to its rarity and varied presentations, necessitating a comprehensive diagnostic approach. Initial investigations often include ultrasound to assess for gallstones and gallbladder wall thickening, followed by CT scans for detailed anatomical information. CTA is invaluable for identifying vascular abnormalities and pinpointing the bleeding vessel (7). Esophagogastroduodenoscopy (EGD) is essential to exclude other UGIB etiologies and may reveal blood emanating from the ampulla of Vater. Endoscopic retrograde cholangiopancreatography (ERCP) offers direct visualization of the biliary tree, facilitating stone removal or stent placement, and can potentially identify bleeding points.

Peroral cholangioscopy (POC), as employed in this case, represents a significant advancement in the direct visualization of the intrahepatic and common bile ducts (10). This technique involves passing a miniaturized flexible endoscope through a duodenoscope or directly into the biliary tree, often via a side-viewing duodenoscope or following ERCP access. Its primary diagnostic utility lies in providing direct, magnified visualization of the biliary mucosa, enabling the detection of subtle inflammatory changes, small lesions, or bleeding sources that might be missed by conventional imaging or even ERCP. Indications for POC include the evaluation of indeterminate biliary strictures, suspected biliary tumors, and unexplained biliary bleeding. Its advantages include a high diagnostic yield for mucosal lesions, minimal invasiveness compared to surgical exploration, and the potential for targeted biopsies. However, limitations include the need for specialized equipment and expertise, potential complications such as cholangitis or pancreatitis, and its unsuitability for all patients or all bleeding sources, particularly those originating from larger extrabiliary vessels. While cholangioscopy has been utilized in the evaluation of biliary diseases, its specific application in diagnosing gallbladder hemorrhage secondary to impacted stones and necrosis appears to be rare in the literature. To our knowledge, this case represents one of the earliest reports documenting the successful diagnosis of hemobilia originating from a bleeding cystic artery due to gallbladder necrosis, precisely identified and confirmed using POC. The POC in this patient revealed a clear bleeding point associated with necrotic mucosa at the gallbladder neck, directly supporting the diagnosis and guiding subsequent management.

Management strategies for hemobilia are tailored to the underlying cause, severity of bleeding, and patient stability. Conservative management, including bowel rest and supportive care, may be considered for mild cases but often fails to address the root pathology. Endoscopic interventions, such as ERCP for stone removal or dilation of strictures, can be effective for specific causes. Transarterial embolization (TAE) offers a minimally invasive option for controlling active arterial bleeding, particularly in cases of pseudoaneurysms or when surgical intervention is high-risk. Radiological interventions, including TAE, have shown efficacy in managing iatrogenic hemobilia (8). Radiofrequency ablation (RFA) has also been explored for hemobilia treatment (11). For more severe or persistent bleeding, surgical management, typically cholecystectomy, is indicated for definitive treatment, especially when significant gallbladder pathology, recurrent bleeding, or failure of less invasive methods is present. Endoscopic hemostasis with self-expandable metal stents has also been utilized as a bridge therapy (12).

In this patient, given the unequivocal evidence of gallbladder necrosis and the identified active bleeding source at the gallbladder neck, direct laparoscopic cholecystectomy was deemed the most appropriate definitive management strategy. While TAE could have potentially controlled the immediate hemorrhage, it would not have addressed the underlying necrotic gallbladder, which posed a persistent risk of inflammation and future complications. ERCP, while valuable for diagnosis and stone management, is not the primary treatment for a bleeding, necrotic gallbladder. Considering the patient's relative hemodynamic stability at the time of decision-making and the clear localization of the bleeding source within the gallbladder, laparoscopic cholecystectomy offered definitive treatment by removing the diseased organ and controlling the hemorrhage at its origin. This approach circumvented the risks associated with prolonged conservative management or staged interventions, providing a complete resolution of the hemobilia.

## Conclusion

In summary, this case highlights the significant diagnostic and therapeutic value of cholangioscopy in managing biliary hemorrhage, providing clinicians with new insights and methodologies. It also underscores the importance of multidisciplinary collaboration in the management of rare diseases. While further refinement of the instrumentation is needed, the use of the eyemax cholangioscope represents a viable option for the diagnosis and treatment of biliary hemorrhage.

## Data Availability

The raw data supporting the conclusions of this article will be made available by the authors, without undue reservation.
